# Editorial: Rodent model organisms: therapeutic treatments and drugs interaction with the gut microbiome

**DOI:** 10.3389/fmicb.2025.1581166

**Published:** 2025-04-01

**Authors:** Eugenia Bezirtzoglou, Julio Plaza-Diaz, Jiajia Song, Guoxiang Xie, Elisavet Stavropoulou

**Affiliations:** ^1^Laboratory of Hygiene and Environmental Protection, Department of Medicine, Democritus University of Thrace, Alexandroupolis, Greece; ^2^School of Health Sciences, Universidad Internacional de La Rioja, Logroño, Spain; ^3^College of Food Science, Southwest University, Chongqing, China; ^4^Human Metabolomics Institute, Shenzhen, China

**Keywords:** gut microbiome, drugs, therapy, mouse, rat, model

## Gut microbiome and health

The microbiome plays a crucial role in our health, unlocking microbial ecosystems (Stavropoulou et al., 2021a). Microbiome research is a dynamic and evolving field that investigates microbial communities and their interconnections (Stavropoulou et al., 2021b).

The interactions of the human microbiome, particularly the gut microbiome, has been extensively studied due to its substantial impact on health (Tsigalou et al., 2018). Research shows that intestinal microbes participate in digestive processes, synthesize vitamins and enzymes, and regulate immunity, inflammatory processes, and microbial balance (Fu et al., 2023; Mangan et al., 2018). Breakdown of microbial balance is associated with multiple disease states, such as diabetes (Li et al., 2020), obesity (Aoun et al., 2020), autoimmune diseases (Mousa et al., 2022), periodontitis (Abdulkareem et al., 2023), stress (Guan et al., 2017), while it can significantly affect drug metabolism (Dodd and Cann, 2022) and individual patient responses (Ma et al., 2024).

Microbiome-based therapies (Yadav and Chauhan, 2022), such as probiotics (Stavropoulou and Bezirtzoglou, 2020), prebiotics (Yadav and Chauhan, 2022), and fecal microbiome transplantation (Liu X. et al.) influence the microbiome and favorably balance it in clinical applications. Personalized medicine (Kashyap et al., 2017) may soon harness microbiome-tailored therapies, but bioethical issues, such as microbiome ownership and data privacy, require attention. Despite the progress in microbiome research, challenges still remain, as microbiomes depend on many factors, such as diet, genetics, lifestyle, and environment (Dong and Gupta, 2019). Rodent model organisms, notably mice and rats, are widely used in microbiome research to investigate therapeutic applications and drug interactions within the gut ecosystem (Vandamme, 2014).

Microbiome research is often conducted on rodents rather than humans for several primary reasons. Studies in rodents permit research to be conducted in a regulated and controlled setting that excludes specific factors. Rodents have short life spans and reproduce quickly. However, they have physiological and genetic similarities in common with humans, making them ideal models for investigating immune responses, metabolic diseases and drug interactions. This is in contrast to human clinical studies, which require long-term monitoring and ethical considerations (Vandamme, 2014; Bryda, 2013).

With this in mind, the present Research Topic focused on publishing research that explores microbial ecosystems in the vertebrate digestive tracts.

Researchers have conducted numerous studies to improve understanding of the field. Certainly, advancements in sequencing technologies, such as metagenomics and 16S rRNA sequencing, have enabled scientists to study microbiomes with unprecedented precision. These innovative methods facilitate the identification of microbes, their genetic functions, and their interconnections within ecosystems (Satam et al., 2023; Kim et al., 2024).

This Research Topic highlights studies that focus on disruptions in microbial composition, resulting in dysbiosis, inflammation, and metabolic disorders.

Extended research into natural products, probiotics and prebiotics was conducted in China with rodent models to evaluate their role in restoring microbial balance. Traditional natural products, such as Gynura segetum root (GSrE) (Gu et al.), tuina herbal (Wang H. et al.), Sanwei sandalwood (Ma K. et al.), Ziyan green tea (Jia et al.), Scrophulariae radix-Atractylodes sinensis (Guo X. et al.), Sheng-Jiang powder (Zhang P. et al.), Banxia-Yiyiren (Wang, Qi et al.), Gegen Qinlian (Xu et al.), Liquiritigenin (Suo et al.), Xiaojin pill (Yang, Quan et al.), Schisanlactone (Song et al.), Liqi Tongbian (Liu Q. et al.), Shengu granules (Chen et al.), Evodiae fructus (Liang et al.), and Bazi Bushen (Zhang S. et al.) were extensively studied in rodent models. Traditional Chinese medicine (TCM) practitioners are becoming more interested in studying the microbiome and how it might be modulated to help treat a variety of illnesses. These TCM herbal formulations can treat metabolic disorders, gastrointestinal inflammatory diseases, autoimmune diseases, and mental and neurological conditions. They do so by modulating the microbial balance, alleviating inflammation, and boosting immune function. Furthermore, fermentation-based TCM formulations that provide bioactive substances contribute effectively to modulating the microbial balance and improving the microbiome. TCM was found to act on intestinal and extraintestinal tissues (Suo et al.). It protected against pathogens by boosting immune cell function and inflammatory responses.

It was also used to relieve insomnia, reduce stress and alleviate anxiety, demonstrating an antidepressant effect through its influence on the gut-brain axis (Wang, Qi et al.). The microbiome-gut-brain axis may also be involved in the pathogenesis and progression of central age-related diseases while it appeared to play a key role in Alzheimer's disease (Song et al.).

TCM was used to treat benign prostatic hyperplasia (Yang, Quan et al.), periodontitis (Guo X.-P. et al.), and colonic cancer by suppressing chronic inflammation (Yang, Quan et al.). Similarly, TCM helped ameliorate non-alcoholic fatty liver disease by alleviating inflammation, regulating lipid metabolism, reducing oxidative stress, and restoring gut microbiome balance, ultimately supporting liver health and improving insulin sensitivity (Zhang P. et al.; Zaparte et al.). However, chronic jet lag disrupted the gut microbiome and mycobiome, contributing to metabolic dysfunction-associated fatty liver disease in mice on a high-fat, high-fructose diet (Zheng et al.).

TCM was also found to play a significant role in managing diabetes by alleviating inflammation, regulating blood glucose levels, improving insulin sensitivity, and balancing the gut microbiome (Hou et al.). Single-anastomosis duodenal–ileal bypass with sleeve gastrectomy appeared to modify gut microbiome composition and thus glucose metabolism in rats with type 2 diabetes (Wang, Li et al.). Metformin has been linked to shifts in gut bacteria, notably increasing *Akkermansia muciniphila* and short-chain fatty acid-producing bacteria. These changes helped stabilize the gastrointestinal mucosal barrier, regulate bile acid metabolism, and supported glucose and lipid metabolism, improving glucose homeostasis and reducing inflammation (Wang Y. et al.).

TCM was found to be effective in the treatment of cardiometabolic diseases and to contribute to heart failure management by improving gut microbiome function, reducing inflammation, enhancing nutrient absorption, and restoring microbial balance (Wang Q. et al.). By modulating the gut-heart axis, TCM formulations were found to regulate metabolism, regulate blood pressure, improve lipid metabolism, strengthen cardiac function, and mitigate disease progression (Wang Q. et al.). Additionally, gut ecological dysregulation appeared to be associated with Sugen5416/hypoxia-induced disease development (Abudukeremu et al.). Ang1-7 and MLN-4760 played a key role in the progression of this pathology. The ACE2-Ang-(1-7)-Mas axis modulated blood pressure and inhibited myocardial remodeling (Abudukeremu et al.).

TCM was also used in the management of osteoporosis following ovariectomy by promoting bone health, balancing hormones, and improving circulation by modulating the gut-bone immune axis (Chen et al.). However, there are concerns about the potential toxicity of TCM due to the concentration of active compounds and the risk of hepatotoxicity (Suo et al.; Liang et al.). Some herbs, when used inappropriately or in excessive amounts, may cause liver damage or interact negatively with other medications. It is imperative to monitor the dosage, quality, and source of herbal preparations, and to consult with healthcare providers. This is to ensure safe and effective use. The gut microbiome appears to play a role in various pathophysiological processes in diabetic ischemic heart failure. It does so by disrupting metabolism and influencing downstream signaling pathways through their metabolites. These gut microbiome and serum metabolites may serve as markers of myocardial damage in different stages of diabetic ischemic heart failure (Hou et al.; Wang Q. et al.).

TCM showed beneficial effect on conditions such as inflammatory diseases (López-Cauce et al.) and slow-transit constipation (Liu Q. et al.) as several TCM formulations reduced inflammation, regulated bowel movements, and balanced the digestive system, alleviating symptoms such as abdominal pain and diarrhea. However, *Akkermansia* deficiency and mucin depletion were found to be implicated in intestinal barrier dysfunction as early events in the development of inflammation in interleukin-10-deficient mice (López-Cauce et al.).

Electroacupuncture is an advanced variation of traditional acupuncture (Yue et al.), in which small electrical currents are passed through acupuncture needles for therapeutic purposes. It is used to treat chronic pain, muscle tension, stress, digestive diseases, diabetes (Yue et al.), and neurological conditions, as electrical boosting was found to improve vascular angiogenesis (Huang et al.), blood flow (Huang et al.), reduce inflammation (Huang et al.), and regulate the nervous system for pain relief, but also appeared to modify the urinary metabolome and microbiome (Gao et al.).

Oxygen is a key component of the air we breathe and plays a critical role in cellular respiration. One study has investigated the positive effects of oxygen enrichment on the structure and composition of the gut microbiome in animals subjected to acute hypobaric hypoxia (Ma Q. et al.).

Probiotics and prebiotics were also tested in rodent models to evaluate their role in restoring microbial balance. The alleviating effect of *Lactobacillus rhamnosus* SDSP202418 on exercise-induced fatigue in mice was reported by Yang et al.(a) while the protective effects of *Lactococcus lactis* subsp. *lactis* HFY14 supplementation appeared to positively impact the brain, gut, and motor function of antibiotic-treated mice [Yang et al.(b)]. Furthermore, a study on the effect and mechanism of *Lacticaseibacillus rhamnosus* AFY06 on inflammation-associated colorectal cancer induced by azoxymethane/dextran sulfate sodium in mice showed promising results (Zhang J. et al.).

Heat acclimation with probiotics-based oral rehydration salts supplementation was found to alleviate heat stroke-induced multiple organ dysfunction by improving intestinal thermotolerance and modulating gut microbiome in rats (Li et al.).

Dysfunctions in intestinal microorganisms and enzyme activities suggest a potential role in the diarrhea of kidney-yang deficiency syndrome. Specifically, a decrease in *Lactobacillus* and *Bifidobacterium* was noted, associated with an increase in *Escherichia coli* (Zhou M. et al.).

Another round of manuscripts focused on the influence of the gut microbiome on drug metabolism. Microbes can enhance, diminish, or alter the effectiveness of a drug, potentially leading to unexpected side effects. These insights contribute to refining drug development and advancing personalized treatment strategies (Wang Y. et al.; Wang Q. et al.). Folic acid and zinc were found to ameliorate hyperuricemia by inhibiting uric acid biosynthesis and stimulating uric acid excretion by modulating the gut microbiome. Thus, folic acid and zinc may be new and safe therapeutic agents to improve hyperuricemia (Sun et al.).

Study findings have identified the preferential distribution of transcription factor EB in colonic epithelial cells, where transcription factor EB can be activated by infection to enhance anti-bacterial peptide expression, holding promising implications for anti-bacterial therapeutics (Rao et al.). The transcription factor EB has significant implications for drug metabolism, particularly in the colon, where microbial interactions influence drug bioavailability and therapeutic outcomes (Rao et al.).

The cathelicidin-related antimicrobial peptide was found to play a critical role in innate immunity and gut homeostasis. Recent research has uncovered its therapeutic potential in alleviating acute ulcerative colitis (Jiang et al.).

Sigma-1 knockout was found to disrupt the gut microbiome and serum metabolome, leading to altered metabolic pathways that exacerbate heart failure. By influencing key metabolic processes and increasing systemic inflammation, this disruption was observed to worsen cardiac function (Yang J.-Z. et al.). Targeting metabolism, particularly through restoring gut microbiome balance and regulating metabolic pathways, may offer potential therapeutic strategies for mitigating heart failure in Sigmar1-deficient models (Yang J.-Z. et al.). Gut microbes were found to regulate the expression of key metabolic enzymes and transporters such as CYP3A1, UGT1A1, and P-GP, all of which are involved in the absorption and clearance of Cyclosporine A, thereby influencing its pharmacokinetic profile (Zhou J. et al.). Research in rodent models showed that broad-spectrum antibiotics can severely alter microbial diversity, resulting in dysbiosis, heightened vulnerability to infections, and the development of metabolic imbalances (Parodi et al., 2022).

As already discussed, dietary choices can directly influence the composition and activity of the gut microbiome. Both food intake and fasting were found to influence gut metabolic processes, immune function, and even the ability to resist infection, highlighting the critical role of diet and fasting in shaping microbiome health (Dong and Gupta, 2019; Frias et al.).

In conclusion, rodent models have proven invaluable in advancing our understanding of the complex interactions between the gut microbiome and various therapeutic options. These models have provided significant insights into how microbial ecosystems influence drug metabolism, immune responses, metabolic diseases, and the progression of various conditions. Research has highlighted the substantial impact the gut microbiome can have on the pharmacokinetics of drugs, such as Cyclosporine A. It has also shown that modifying specific microbial communities can significantly impact drug efficacy and patient outcomes. Furthermore, traditional medicine, especially within the context of TCM, offers promising therapeutic approaches that modulate the gut microbiome for the management of metabolic conditions, autoimmune diseases, and even neurodegenerative conditions, further emphasizing the importance of gut health.

Although promising, challenges remain in understanding the full scope of microbial contributions to drug metabolism and disease progression. More research is needed to fully elucidate their underlying mechanisms. Furthermore, potential risks associated with microbiome-based therapies, such as toxicity and drug-drug interactions, must be carefully addressed to ensure safe and effective application in clinical settings. Ultimately, the gut microbiome represents a central area of research with transformative potential in personalized medicine. It offers new strategies to optimize therapeutic interventions and improve patient health outcomes.
